# *Borrelia valaisiana* Resist Complement-Mediated Killing Independently of the Recruitment of Immune Regulators and Inactivation of Complement Components

**DOI:** 10.1371/journal.pone.0053659

**Published:** 2013-01-08

**Authors:** Jasmin Schwab, Claudia Hammerschmidt, Dania Richter, Christine Skerka, Franz-Rainer Matuschka, Reinhard Wallich, Peter F. Zipfel, Peter Kraiczy

**Affiliations:** 1 Institute of Medical Microbiology and Infection Control, University Hospital of Frankfurt, Frankfurt, Germany; 2 Abteilung Parasitologie, Institut für Pathologie, Charité Universitätsmedizin Berlin, Berlin, Germany; 3 Department of Infection Biology, Leibniz Institute for Natural Product Research and Infection Biology, Jena, Germany; 4 Institute of Immunology, University of Heidelberg, Heidelberg, Germany; 5 Friedrich Schiller University, Jena, Germany; Institute of Medical Microbiology and Hospital Epidemiology-Hanover Medical School, Germany

## Abstract

Spirochetes belonging to the *Borrelia (B.) burgdorferi* sensu lato complex differ in their resistance to complement-mediated killing, particularly in regard to human serum. In the present study, we elucidate the serum and complement susceptibility of *B. valaisiana*, a genospecies with the potential to cause Lyme disease in Europe as well as in Asia. Among the investigated isolates, growth of ZWU3 Ny3 was not affected while growth of VS116 and Bv9 was strongly inhibited in the presence of 50% human serum. Analyzing complement activation, complement components C3, C4 and C6 were deposited on the surface of isolates VS116 and Bv9, and similarly the membrane attack complex was formed on their surface. In contrast, no surface-deposited components and no aberrations in cell morphology were detected for serum-resistant ZWU3 Ny3. While further investigating the protective role of bound complement regulators in mediating complement resistance, we discovered that none of the *B. valaisiana* isolates analyzed bound complement regulators Factor H, Factor H-like protein 1, C4b binding protein or C1 esterase inhibitor. In addition, *B. valaisiana* also lacked intrinsic proteolytic activity to degrade complement components C3, C3b, C4, C4b, and C5. Taken together, these findings suggest that certain *B. valaisiana* isolates differ in their capability to resist complement-mediating killing by human serum. The molecular mechanism utilized by *B. valaisiana* to inhibit bacteriolysis appears not to involve binding of the key host complement regulators of the alternative, classical, and lectin pathways as already known for serum-resistant Lyme disease or relapsing fever borreliae.

## Introduction

Lyme disease, the most prevalent vector-borne anthropozoonosis in Europe and North America is caused by spirochetes belonging to the *Borrelia (B.) burgdorferi* sensu lato (s.l.) complex [1]. In Central Europe, *B. burgdorferi*, *B. afzelii*, *B. garinii*, *B. spielmanii* as well as *B. bavariensis* are the causative agents of Lyme disease while the pathogenic potential for *B. valaisiana* remains unclear [2]–[6]. There are several lines of evidence that *B. valaisiana* (formely described as genomic groups VS116 and M19) might also cause human Lyme disease even though only skin biopsies or cerebrospinal fluid samples of erythema migrans, acrodermatits chronica atrophicans, and neuroborreliosis patients were found to be positive for *B. valaisiana* DNA [3], [4]. Complement plays an important role for the recognition, discrimination, and elimination of invading microorganisms [7]. As a central part of the human innate immune system, it is immediately activated upon entry of an intruder via the alternative or the lectin pathways and also by the classical pathway. Activation results in the cleavage of the central component C3 and the generation of active splice fragments C3a and C3b, and deposition of highly-reactive C3b molecules on activator cell surfaces leads to opsonization followed by opsonophagocytosis. In addition, progression of the cascade and formation of the lytic membrane attack complex (MAC) results in complement-mediated killing of the invading pathogen.

The potent complement regulators include C1 esterase inhibitor (C1-Inh), a highly glycosylated molecule of approximately 105 kDa [8] which regulates the initial complement activation steps of the classical and lectin pathways [9]–[11]. Interaction of C1-Inh with C3b and factor B interferes with formation of the C3 convertase and thereby also prevents alternative pathway activation [11].

Factor H (CFH) and Factor H-like protein 1 (FHL1) are the key inhibitors of the alternative pathway, both of which act as co-factors for factor I-mediated inactivation of C3b to iC3b, inhibiting the formation and accelerating the decay of the C3bBb convertase, and finally competing with factor B for binding to C3b [12]–[15]. CFH is composed of 20 individually folding protein domains, termed short complement regulator (SCR) [15]. The 42-kDa FHL1 protein consists of the seven N-terminal SCRs of CFH, including the complement regulatory domains and has a unique C-terminal extension of four hydrophobic amino acid residues [15]. In addition, there are six Factor H-related proteins (CFHRs) all of which belong to the human CFH family [16], [17]. A common feature of these molecules is the high degree of similarity of their C-terminal SCR domains of CFH [16], [18]. CFHR1 acts as a complement regulator by inhibiting the assembly and membrane insertion of the terminal membrane attack complex and inhibits C5 convertase activity [19] while the biological function(s) of CFHR2 is as yet unclear.

The key fluid-phase complement regulator of the classical pathway, C4Bp acts as cofactor for factor I-mediated degradation of C4b to C4d and facilitates dissociation of C2a from the C3 convertase C4b2a of the classical pathway. By preventing formation of the C3 convertase, the generation of C5b and further downstream activation products in the cascade are impeded [20], [21].

Differences in host specificity largely correlate with the resistance/sensitivity pattern of Lyme disease spirochetes to complement and allow the various kinds of borreliae to selectively survive in diverse mammalian hosts [22], [23].

The most prominent complement evasion strategy used by serum-resistant borreliae involves binding of host-derived fluid-phase complement regulators CFH and FHL1 [24]–[27]. While elucidating the molecular mechanisms of complement resistance, two major groups of genetically and structurally unrelated molecules, collectively termed complement regulator-acquiring surface proteins (CRASPs) have been identified among serum-resistant *B. burgdorferi*, *B. afzelii*, *B. spielmanii*, and *B. bavariensis* isolates. These molecules represent ligands for CFH and FHL1 or CFH, CFHR1, CFHR2, and CFHR5 [25], [28]–[31]. The CFH/FHL1-binding proteins consists of CspA (formerly referred to as BBA68 or CRASP-1) of *B. burgdorferi*, *B. afzelii*, and *B. spielmanii*, and CspZ (formerly referred to as BBH06 or CRASP-2) of *B. burgdorferi* [31]–[34]. The CFH/CFHRs-binding proteins include members of the Erp (OspE/F-related) protein family ErpP (CRASP-3), ErpC (CRASP-4), ErpA (CRASP-5), OspE, and the p21 protein [27], [28], [35]–[40]. It has also been shown that serum resistance of *B. burgdorferi* directly correlates with the expression of CspA or CspZ [41]–[43].

To extend our analysis on complement resistance of Lyme disease spirochetes, we examined the serum susceptibility of three *B. valaisiana* isolates derived from feeding or questing *Ixodes ricinus* ticks. Here we show for the first time, that a particular *B. valaisiana* strain resists complement-mediated killing. Interestingly, the molecular mechanism of complement resistance differs from other borrelial species in that it is independent of the recruitment of complement regulators of the alternative (CFH, FHL1), classical (C1-Inh, C4Bp) or Lectin pathways (C1-Inh) or degradation of key complement components C3, C3b, C4, C4b and C5, suggesting that certain spirochetes may have developed alternative strategies to overcome innate immunity.

## Materials and Methods

### Ethics Statement

The study and the respective consent documents were approved by the ethics committee at the Johann Wolfgang Goethe-University of Frankfurt (control number 160/10). All healthy blood donors provided written informed consent.

### Bacterial Strains and Culture Conditions

Borrelial strains *B. burgdorferi* LW2 (skin isolate, Germany), *B. garinii* G1 (CSF isolate, Germany), *B. valaisiana* Bv9 (tick isolate, Germany), *B. valaisiana* ZWU3 Ny3 (tick isolate, Germany), and type strain *B. valaisiana* VS116 (tick isolate, Switzerland) were cultured until mid-exponential phase (5×10^7^ cells per ml) at 33°C in Barbour-Stoenner-Kelly (BSK-H) medium (Bio & SELL, Feucht, Germany). Relapsing fever spirochete *B. duttonii* strain LA1 was cultured at 33°C in BSK-H medium supplemented with 3% of rabbit serum at 33°C. The density of spirochetes was determined using dark-field microscopy and a Kova counting chamber (Hycor Biomedical, Garden Grove, CA).

### Human Sera, Monoclonal and Polyclonal Antibodies, and Human Serum Proteins

Normal human serum (NHS) used for serum susceptibility testing and ligand affinity blotting or as a source of CFH was tested for the presence of anti-*Borrelia* IgM and IgG antibodies by commercially available ELISAs (Enzygnost® Borreliosis/IgM and Enzygnost® Lyme link VlsE/IgG, Siemens Healthcare Diagnostics Products GmbH, Marburg, Germany). Only sera proven to be negative for IgM or IgG anti-*Borrelia* antibodies were used to form the serum pool.

Purified human complement components CFH, C4b binding protein (C4Bp), C1q esterase inhibitor (C1-Inh), C3, C3b, C4, C4b, C5, and Factor I were purchased from Complement Technology, Tyler, TX, USA. The cloning, expression, and purification of FHL1 has been described previously [12]. Polyclonal goat anti-CFH antiserum (dilution 1/1,000), polyclonal anti-C4 antiserum (dilution 1/1,000) (recognizing the α-, β-, and γ-chain as well as the α ´4 fragment but not C4c and C4d), goat anti-human C3 (dilution 1/1,000 for immunofluorescense microscopy and 1/2,000 for Western blotting) (recognizing the α- and β-chain, the α ´68, α ´43 as well as the α ´41 fragment but not C3d) and C6 antibodies (dilution 1/50) were purchased from Merck Biosciences, Bad Soden, Germany. The polyclonal sheep anti-C4Bp antiserum (dilution 1/1,000) was purchased from The Binding Site, Schwetzingen, Germany. Polyclonal anti-C1-Inh antiserum (dilution 1/1,000), the polyclonal anti-C5 antibody (dilution 1/1,000) (recognizing the α- and β-chain), and the monoclonal anti-human C5b-9 antibody (dilution 1/10) were purchased from Quidel (San Diego, CA, USA). Polyclonal rabbit anti-SCR1-4 antiserum was used for detection of CFH and FHL1 [12]. Rabbit polyclonal anti-CFHR1 antibody was used for detection of CFHR1 and CFHR2 and CFHR5 [44].

### Molecular Genotyping of Borrelial Isolates by Restriction Fragment Length Polymorphism

Genotyping of borrelial isolates was performed by PCR amplification of the *ospA* gene followed by restriction fragment length polymorphism analysis as described by Michel et al. [45]. For differentiation, PCR-generated *ospA* fragments were digested separately with 0.5 U of restriction endonucleases BglII, SspI, HindIII (New England Biolabs, Frankfurt, Germany), Kpn21 (Fermentas, St. Leon-Rot, Germany), and SfuI (Roche Applied Science, Mannheim, Germany) overnight according to the manufactureŕs instruction. The digested PCR fragments were then analyzed by electrophoresis on a 2% agarose gel and visualized with ethidium bromide. Fragment size was determined by comparison to DNA fragments of a 123 bp molecular standard (Invitrogen, Karlsruhe, Germany). Genospecies’ designation of the isolates was performed by analyzing the resulting genospecies-specific PCR-RFLP patterns according to Michel et al. [45]. In addition, amplicons generated were sequenced by the dideoxy chain-termination method and the processed sequences were subjected to BLAST search (http://blast.ncbi.nlm.nih.gov).

### Ligand Affinity Blot Analysis

Whole cell lysates obtained from each borrelial isolate (15 µg per lane) were subjected to 10% tris/tricine SDS-PAGE under reducing conditions and transferred to nitrocellulose as previously described [30], [44].

### Serum Susceptibility Testing

Serum susceptibility of borrelial strains was assessed by employing a growth inhibition assay as previously described [46], [47]. Briefly, highly motile spirochetes (1.25×10^7^) diluted in a final volume of 100 µl in BSK medium containing 240 µg ml^−1^ phenol red were incubated with 50% NHS or 50% heat-inactivated human serum (hiNHS) in microtiter plates for 9 days at 33°C (Costar, Cambridge, MA, USA). Growth of spirochetes was monitored by measuring a color shift of the pH indicator in the medium at 562/630 nm using an ELISA reader (PowerWave HT, Bio-Tek Instruments, Winooski, VT, USA). The Gene 5 software was used to calculate the growth curves (Bio-Tek Instruments, Winooski, VT, USA).

### Binding of Complement Proteins to Viable Spirochetes

Borreliae (2×10^9^ cells) grown to mid-log phase were gently washed and resuspended in 750 µl NHS supplemented with 34 mM EDTA (pH 8.0) to avoid complement activation or 750 µl hiNHS. After 1 h incubation at room temperature and four wash steps with PBSA (0.15 M NaCl, 0.03 M phosphate, 0.02% sodium azide, pH 7.2) containing 0.05% Tween-20, proteins bound to the borrelial surface were eluted by incubation with 100 mM glycine-HCl (pH 2.0) for 15 min. Cells were then removed by centrifugation at 14.000×g for 10 min at 4°C, and the supernatant was neutralized by adding 1 M Tris (pH 9.0). Both, the last wash and the eluate fraction were separated by Laemmli-SDS-PAGE under non-reducing conditions and binding of CFH, FHL1, CFHR1, CFHR2, and CFHR5 was analyzed by Western blotting using specific antisera [44]. For C4Bp detection, the samples were separated by 10% tris/tricine SDS-PAGE under reducing conditions.

### Binding of Purified Complement Proteins to Spirochetes by ELISA

Spirochetes grown to mid-log phase were gently washed and resuspended in 500 µl Dulbecco’s PBS without Ca^2+^ and Mg^2+^. Microtiter plates (Polysorb, Nunc, Roskilde, Denmark) were coated with 5×10^7^ cells over night at 4°C. Microtiter plates were washed threefold with PBS containing 0.05% Tween 20 and treated for 1 h at RT with blocking buffer (Applichem GmbH, Darmstadt, Germany). After washing, CFH or C4Bp (500 ng each in a final volume of 100 µl) was added and incubated for 1 h at RT. Thereafter, the wells were washed with PBS containing 0.05% Tween 20 (CFH and C4Bp) and bound CFH or C4Bp was detected with either goat anti-CFH polyclonal antiserum or sheep anti-C4Bp polyclonal antiserum, respectively. After washing, bound proteins were identified using appropriate secondary horseradish peroxidase–coupled antisera. Detection was performed with 1,2-phenylenediamine dihydrochloride as substrate (OPD, Sigma-Aldrich, Taufkirchen, Germany) and absorbance was measured at 490 nm.

### Determination of Intrinsic C3b and C4b Cleavage Activity of Spirochetes

Proteolytic activity of borrelial cells was analyzed in the presence and absence of complement regulators CFH and C4Bp. Cells (5×10^8^) grown to mid-log phase were gently washed and resuspended in either 100 µl hiNHS, 100 µl GVB^++^-buffer (Complement Technology, Tyler, TX, USA) containing 5 µg CFH or 100 µl GVB^++^-buffer containing 5 µg C4Bp, respectively. After incubation for 60 min at room temperature, cells were washed and resuspended in GVB^++^-buffer containing C3b (1 µg/µl) and Factor I (250 ng/µl) or C4b (1 µg/µl) and Factor I (250 ng/µl), respectively. The reaction mixtures were incubated for 60 min at 37 °C and cells were sedimented by centrifugation at 14.000×g for 10 min at 4°C. Each supernatant was then mixed with sample buffer, subjected to SDS-PAGE under reducing conditions and transferred onto a nitrocellulose membrane. C3b and C4b degradation products were visualized by Western blotting using a polyclonal goat anti-C3 antiserum or a polyclonal goat anti-C4 antiserum.

### Determination of Intrinsic C3, C4 and C5 Cleavage Activity of Spirochetes

Spirochetes (5×10^8^) grown to mid-log phase were gently washed and resuspended in 20 µl GVB^++^-buffer (Complement Technology, Tyler, TX, USA) containing 1 µg C3, 1 µg C4 or 1 µg C5. After incubation for 60 min or 120 min at 37 °C with gentle agitation, cells were sedimented by centrifugation at 14.000×g for 10 min at 4°C. Each supernatant was then mixed with sample buffer, subjected to 10% tris/tricin SDS-PAGE under reducing conditions and transferred onto a nitrocellulose membrane. C3, C4 and C5 degradation products were visualized by Western blotting using a polyclonal goat anti-C3 antiserum, a polyclonal goat anti-C4 antiserum or a polyclonal goat anti-C5 antiserum.

### Detection of Deposited Complement Components by Immunofluorescence Microscopy

For detection of complement components deposited on the borrelial surface after complement activation, an immunofluorescence assay was performed as previously described [29], [43]. In brief, spirochetes (6×10^6^) were incubated in 25% NHS or, as a control, in 25% hiNHS for 30 min at 37°C with gentle agitation. Ten microliters of cell suspension were spotted on glass slides, allowed to air dry overnight, and fixed in methanol. After 1 h incubation at 37°C with polyclonal antibodies directed against the complement components C3, C4, and C6 or a mAb directed against the membrane attack complex (MAC) slides were washed and subsequently incubated with Alexa 488-conjugated antibodies directed against either goat or mouse antibodies (Molecular Probes, Eugene, OR, USA). After washing, the slides were mounted with ProLong Gold antifade reagent (Molecular Probes, Eugene, OR, USA) containing DAPI.

### Determination of the C3a and C4a Generation

Spirochetes (6×10^6^) grown to mid-log phase were gently washed and resuspended in 100 µl sample dilution buffer containing 25 µl NHS. After incubation for 5 min at 21°C (C3a) or for 5 min at 37°C (C4a), cells were sedimented by centrifugation at 14.000×g for 10 min at 4°C. As a control, NHS was also pre-incubated for 5 min at 21°C and 37°C, respectively. Supernatants were then diluted as recommend by the manufacturer and samples were applied to microtiter plates containing immobilized monoclonal antibodies against C3a and C4a, respectively. Generation of C3a was analyzed by the MicroVue C3a Plus ELISA (Quidel, San Diego, CA, USA) and for determination of C4a in NHS, the BD OptEIA™ Human C4a ELISA Kit (BD Biosciences, Heidelberg, Germany) was used.

## Results

### Serum Susceptibility of *B. valaisiana*

**Figure 1:**
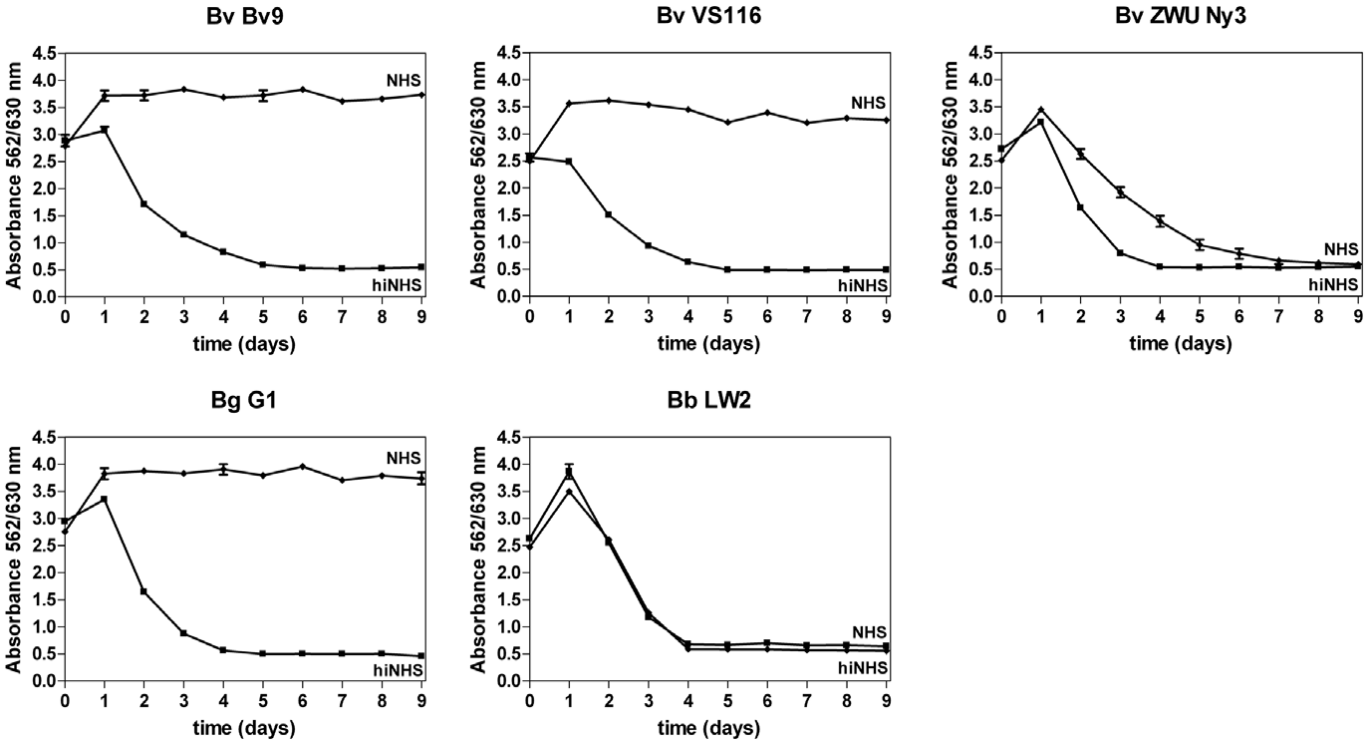
Serum susceptibility testing of *B. valaisiana*. A colorimetric growth survival assay was used to investigate susceptibility to human serum of *B. valaisiana* Bv9, VS116, ZWU3 Ny3, *B. garinii* G1, and *B. burgdorferi* LW2. Spirochetes were incubated in either 50% NHS (diamonds) or 50% hiNHS (rectangles) over an incubation period of 9 days at 33°C. Color changes were monitored by measurement of the absorbance at 562/630 nm. All experiments were performed three times with at least three replicates, obtaining very similar results. For clarity, data of a representative experiment are shown. Error bars represent ± SD.

To further evaluate the serum resistance pattern of *B. valaisiana* to human complement, three isolates collected from ticks at different geographical locations were incubated in 50% active NHS or in 50% hiNHS for up to 9 days and cell growth was monitored in a colorimetric growth survival assay [47], [48]. Growth of viable spirochetes results in a continuous decrease of the absorbance values due to accumulation of secondary metabolites in the BSK-H medium. As demonstrated in figure 1, growth of *B. valaisiana* isolate Bv9, *B. valaisiana* type strain VS116, and serum-sensitive control strain *B. garinii* G1 when challenged with 50% NHS was completely inhibited as evidenced by only minor changes of absorbance values. Under identical experimental conditions, *B. valaisiana* ZWU3 Ny3 as well as serum-resistant *B. burgdorferi* LW2 showed growth in NHS as indicated by continuous decrease of absorbance values. As expected, hiNHS did not affect growth of the five borrelial strains analyzed. In contrast to previous findings, only *B. valaisiana* strain ZWU3 Ny3 resist complement-mediated killing by human serum.

### Deposition of Activated Complement Components on the Surface of *B. valaisiana*

**Figure 2:**
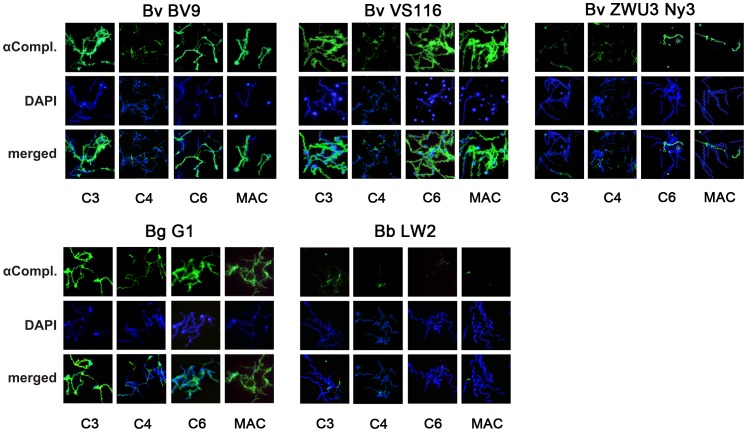
Determination of activated complement components on the surface of *B. valaisiana.* Complement components C3, C4, C6 and MAC deposited on the surface of *B. valaisiana* isolates Bv9, VS116, ZWU3 Ny3, *B. garinii* G1, *B. burgdorferi* LW2 were visualized by indirect immunofluorescence microscopy. Spirochetes were incubated with 25% NHS for 30 min at 37°C with gentle agitation and deposited C3, C4, C6, and MAC were analyzed with specific antibodies against each component and appropriate Alexa 488-conjugated secondary antibodies. For visualization of the spirochetes in a given microscopic field, the DNA-binding dye DAPI was used. The spirochetes were observed at a magnification of 100× objective. The data were recorded with a DS-5Mc CCD camera (Nikon) mounted on an Olympus CX40 fluorescence microscope. Each panel is representative of at least 20 microscope fields.

Next, we assessed deposition of activated complement components C3, C6 and the membrane attack complex (MAC) on the surface of *B. valaisiana* isolates Bv9, VS116, and ZWU3 Ny3 as well as serum-resistant *B. burgdorferi* LW2 and serum-sensitive *B. garinii* G1. Complement C3, C4, C6 and MAC (C5b-9) were strongly deposited on the surface of the serum-sensitive *B. valaisiana* Bv9 and VS116 as well as on the serum-sensitive control strain *B. garinii* G1 (Fig. 2). In contrast, serum-resistant strains *B. valaisiana* ZWU3 Ny3 and *B. burgdorferi* LW2 showed marginal or no fluorescent staining for all three complement components analyzed, suggesting that the complement cascade was inhibited at the level of C3 and C4 activation. Slides were also counterstained with DAPI to identify all spirochetes in a given field of view. Most of the complement-positive spirochetes showed extensive bleb formation when exposed to human serum, while a few cells stained negative for complement but were positive in the DAPI stain. Those spirochetes most likely represent “cell ghost” as previously described for serum susceptible *B. garinii* and *B. lusitaniae* strains [43], [49]. When spirochetes were incubated with heat-inactivated NHS, the cell morphology remained completely intact and deposition of complement was not detectable by immunofluorescence microscopy (data not shown). Thus, treatment of spirochetes with active NHS leads to strong complement activation followed by robust deposition of effector components to *B. valaisiana* Bv9 and VS116 but not to serum-resistant isolate ZWU3 Ny3.

### Identification of CFH-binding Proteins in *B. valaisiana*

**Figure 3:**
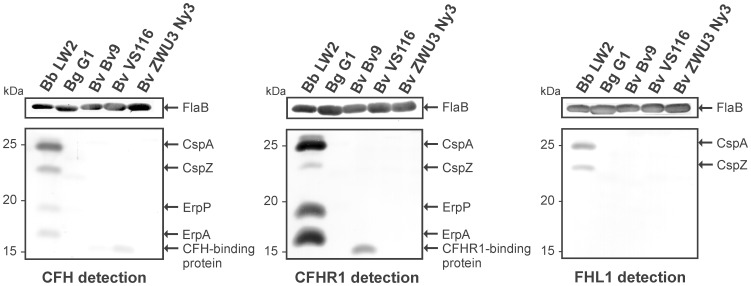
Identification of CRASPs among *B. valaisiana*. Cell lysates (30 µg each) obtained from control strains *B. burgdorferi* LW2 and *B. garinii* G1 as well as from *B. valaisiana* isolates Bv9, VS116, and ZWU3 Ny3 were subjected to 10% tris/tricine SDS-PAGE and transferred to nitrocellulose. The membranes were then incubated with either NHS as source for CFH and CFHR1 or with cell culture supernatant containing recombinant FHL1. Potential CFH/FHL1/CFHR1-binding proteins were detected using a polyclonal anti-CFH serum, a polyclonal anti-CFHR1 serum or a polyclonal anti-SCR1-4 antiserum. Monoclonal antibody L41 1C11 recognizing the FlaB protein was used to show equal loading of bacterial lysates. The identified CRASP proteins, CspA, CspZ, ErpP, and ErpA of *B. burgdorferi* LW2, the CFH-binding protein of *B. valaisiana* Bv9 and VS116, and the CFHR1-binding protein of *B. valaisiana* Bv9 are indicated on the right and the mobility of the marker proteins is indicated on the left.

It is well-established that resistance of *B. burgdorferi*, *B. afzelii*, and *B. spielmanii* to complement-mediated lysis directly correlates with binding of complement regulators CFH and FHL1 [24], [25], [29], [31], [36]. For this reason, we sought to identify potential CFH/FHL1-binding proteins produced by serum-resistant *B. valaisiana* ZWU3 Ny3 using ligand affinity blotting. Whole cell lysates obtained from all three *B. valaisiana* isolates and the two control strains were separated by SDS-PAGE and proteins were transferred to nitrocellulose membranes. Following incubation with NHS as a source for CFH and a polyclonal anti-CFH antiserum, a very faint signal corresponding to a CFH-binding protein of approximately 15 kDa could be detected within serum-sensitive *B. valaisiana* isolate Bv9 and VS116 but not in serum-resistant ZWU3 Ny3 (Fig. 3). Using a polyclonal anti-CFHR1 antiserum, Bv9 but neither VS116 nor ZWU3 Ny3 showed a 15 kDa CFHR1-binding protein. For comparison, a cell lysate obtained from serum-resistant *B. burgdorferi* LW2 showed four CFH-binding CRASP proteins (CspA, CspZ, ErpP, and ErpA), three proteins that strongly bound CFHR-1 (CspA, ErpP and ErpA) and two FHL1-binding proteins (CspA and CspZ). Furthermore, FHL1-binding proteins could not be detected within the three *B. valaisiana* isolates. In agreement with our previous data, serum-sensitive *B. garinii* G1 did not produce any CFH/FHL1-binding proteins under *in vitro* cultivation [25], [29], [43]. Taken together, distinct *B. valaisiana* isolates are able to produce a 15 kDa protein that possesses potential CFH/CFHR1-binding capability, but apparently did not contribute to resistance to complement.

### Identification of Complement Regulators Bound to the Surface of Intact Borrelial Cells

**Figure 4:**
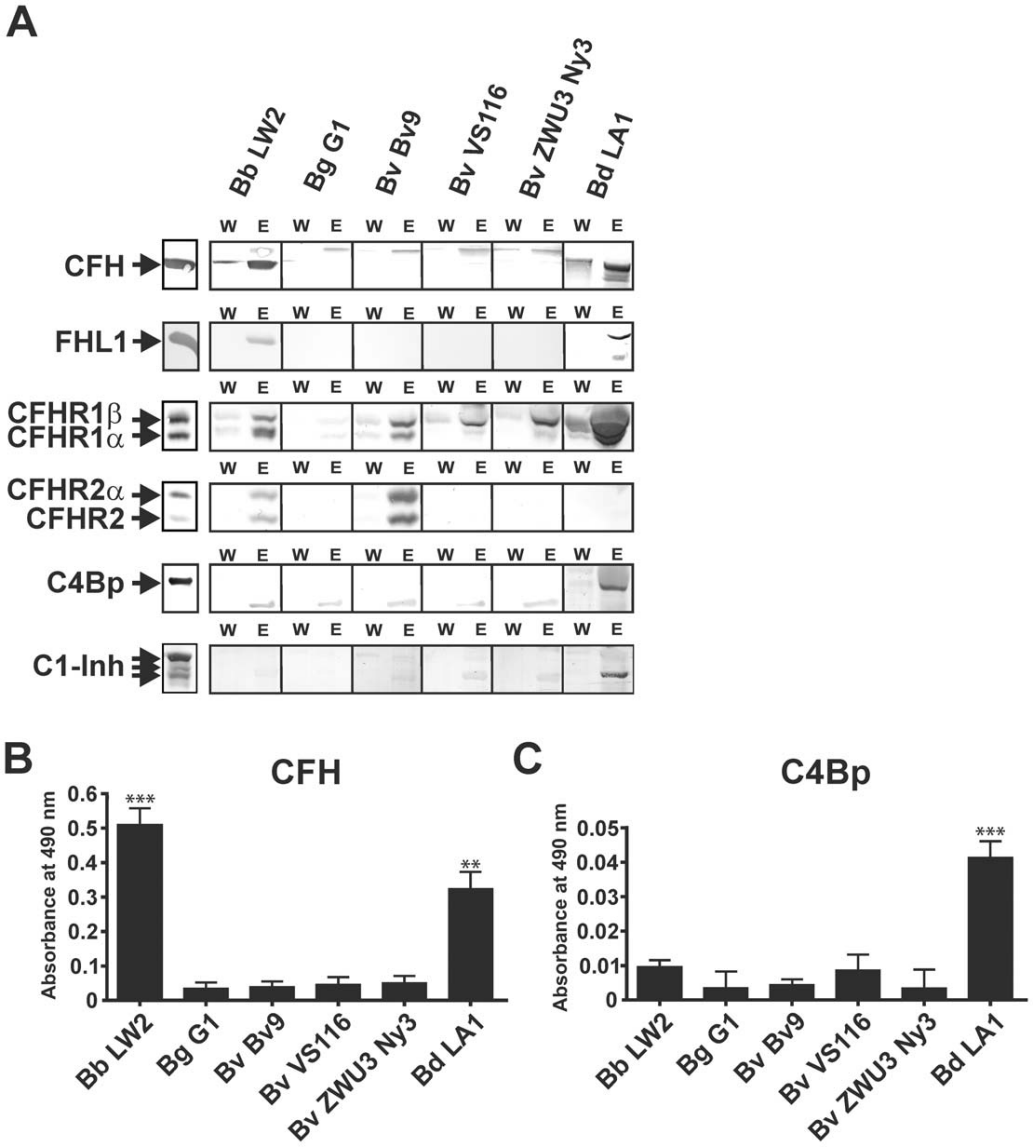
Binding of complement regulators to intact spirochetes. (A) Ligand affinity blotting was performed to detect binding of complement regulators. *B. burgdorferi* LW2, *B. garinii* G1, *B. valaisiana* isolates Bv9, VS116, and ZWU3 Ny3 as well as *B. duttonii* LA1 were incubated in NHS-EDTA to prevent complement activation, washed extensively, and bound proteins were eluted using 100 mM glycine-HCl (pH 2.0). Both the last wash (W) and the eluate (E) fractions obtained from each strain were separated by 12.5% Laemmli-SDS-PAGE (detection of CFH, FHL1, FHR1, FHR2, and C1-Inh) or by 10% tris/tricine SDS-PAGE (C4Bp detection) and transferred to nitrocellulose. Membranes were probed either with a polyclonal anti-SCR1-4 antiserum for the detection of both CFH and FHL1, a polyclonal anti-CFHR1 antiserum for the detection of CFHR1 and CFHR2, a polyclonal anti-C4Bp antiserum, or with a polyclonal anti-C1-Inh antiserum. Binding of CFH (B) or C4Bp (C) to spirochetes (5×10^7^ cells) was assessed by ELISA. Bound CFH was detected with a polyclonal goat anti-CFH antiserum and C4Bp was detected with a polyclonal sheep anti-C4Bp antiserum. Data represent means and SD from three separate experiments, each performed at least in triplicate. ***p<0.001; **p<0.01. Raw data were analyzed by one-way ANOVA with post hoc Bonferroni correction.

Serum-resistant *B. burgdorferi*, *B. afzelii*, and *B. spielmanii* bind complement regulators CFH and FHL1 to circumvent complement-mediated lysis [24], [25], [29]. To further examine whether *B. valaisiana* is able to recruit complement regulators from human serum, borrelial cells were incubated in NHS-EDTA (to avoid complement activation) (Fig. 4). Following extensive washing, bound proteins were eluted from the spirochetal surface using 0.1 M glycine-HCl (pH 2.0). The last wash and the final eluate fraction were collected, separated by SDS-PAGE and subjected to Western blotting using different polyclonal antisera for the detection of complement regulators CFH, FHL1, CFHR1, CFHR2, CFHR5, C4Bp, and C1-Inh. CFH and FHL1, the key regulators of the alternative pathway could be detected in the eluate fraction obtained from serum-resistant control strains *B. burgdorferi* LW2 and *B. duttonii* LA1, but not in the appropriate fractions of serum-sensitive *B. valaisiana* Bv9 and VS116 as well as serum-resistant *B. valaisiana* ZWU3 Ny3. As expected, serum-sensitive control strain *B. garinii* G1 did not bind CFH and FHL1 at all. Of note, the faint band above CFH detected represents cross-reacting immunoglobulins derived from the BSK culture medium.

CFH-binding proteins belonging to the Erp protein family, in particular ErpA/CRASP-5, ErpC/CRASP-4, ErpP/CRASP-3 as well as OspE bound CFH only under denaturated conditions in a solid phase assay (e.g. by using purified recombinant proteins or borrelial cell lysates) but did not bind CFH when the same proteins are exposed on the surface of viable cells [44], [50]. By contrast, these borrelial proteins bound strongly to other members of the CFH protein family, namely CFHR1, CFHR2 and to a lower extent to CFHR5. Elucidating binding of CFHRs to *B. valaisiana* Bv9 and VS116 producing the 15 kDa CFH-binding protein, the wash and eluate fractions of all borrelial isolates tested were also analyzed for CFHR1 and CFHR2 (Fig. 4A). The two glycosylated forms of CFHR1 (CFHR1α and CFHR1β) were detected in the eluate fractions of *B. burgdorferi* LW2 (this strain produced CFHR-binding proteins ErpP and ErpA) as well as *B. valaisiana* Bv9, VS116, and ZWU3 Ny3 but not in the eluate fraction of *B. garinii* G1. Intriguingly, the binding patterns of the 15 kDa proteins of *B. valaisiana* Bv9 and VS116 resemble the CFHR-binding Erp proteins ErpA/CRASP-5 and ErpP/CRASP-3 of *B. burgdorferi* LW2. Binding of CFHR2 and CFHR2α was only detected for *B. burgdorferi* LW2 and *B. valaisiana* Bv9. Concerning the African tick-borne relapsing fever spirochete *B. duttonii* LA1, binding of CFHR1α and CFHR1β could be detected for this particular borrelial strain.

Next, we sought to determine whether *B. valaisiana* is able to bind C4Bp and/or C1-Inh as the key complement regulators of the classical and lectin pathways. To this end, isolates were incubated with NHS-EDTA (for C4Bp binding) or heat-inactivated NHS (for C1-Inh binding). As demonstrated in Figure 4, none of the Lyme disease spirochetes bound C4Bp or C1-Inh to their surface. To exclude possible limitations of our experimental setup, we also investigated *B. duttonii* LA1 known to bind C4Bp and C1-Inh [51], [52]. Employing identical conditions for the serum adsorption assays, binding of C4Bp and C1-Inh to strain LA1 could be clearly detected by Western blotting (Figure 4A).

Interaction of spirochetes with CFH and C4Bp was also assessed by ELISA as quantitative method for analyzing binding of distinct complement regulators. Again, none of the *B. valaisiana* isolates bound to purified CFH and C4Bp (Figures 4B and C, respectively). In addition, binding of CFH and C4Bp to spirochetes were also assessed under less stringent washing conditions. Again, none of the *B. valaisiana* strains including ZWU3 Ny3 bound either complement regulators (data not shown). This finding strongly suggests that isolate ZWU3 Ny3 overcomes complement-mediated killing by a different mechanism that is independent of binding of host-derived complement regulators.

### Determination of the Potential Intrinsic Proteolytic Activity of *B. valaisiana* for Various Complement Components

**Figure 5:**
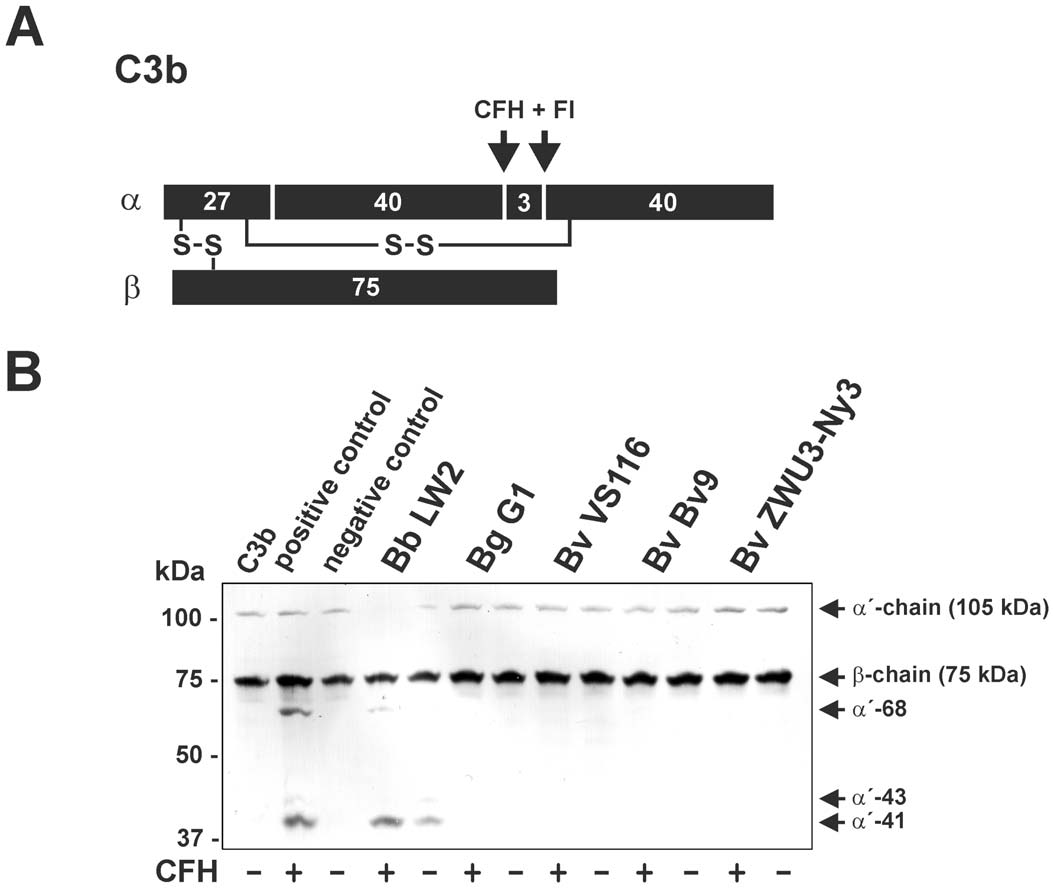
Determination of the C3b proteolytic activity of *B. valaisiana*. (A) Schematic representation of the α- and the β-chain of C3b and the cleavage fragments of the α-chain generated by CFH and Factor I. (B) Degradation of C3b by an intrinsic proteolytic activity of borrelial cells (4×10^7^) was analyzed by detection of characteristic cleavage fragments after incubation of spirochetes with (+) or without (−) purified CFH. *B. burgdorferi* LW2, *B. garinii* G1, *B. valaisiana* isolates Bv9, VS116, and ZWU3 Ny3 were incubated with CFH for 60 min at room temperature. After extensive washing with PBS, C3b (10 ng/ml) and Factor I (20 ng/ml) were added and the mixture was incubated for 30 min at 37°C. Subsequently, samples were heated to 95°C for 5 min, subjected to 12.5% Laemmli-SDS-PAGE and transferred onto a nitrocellulose membrane. The C3b degradation products were visualized by Western blotting using a polyclonal goat anti-human C3 antiserum. As a positive control, purified CFH (50 ng) was incubated with C3b and Factor I, and as a negative control complement proteins were incubated in the absence of CFH. The mobility of the α’- and the β-chain of C3b and the cleavage products of the α’-chain (α’-68 and α’-43) is indicated. (+) incubation with all complement proteins; (−) incubation without CFH.

To clarify our somewhat paradoxical observation that serum-resistant *B. valaisiana* ZWU3 Ny3 failed to bind any of the tested complement regulators to its surface, a degradation assay was employed to detect intrinsic proteolytic cleavage capacity of spirochetes for C3b (Fig. 5). To this end, spirochetes were incubated with or without purified CFH for 60 min. After extensive washing, Factor I and C3b were added to each reaction mixture and following incubation, the reaction mixtures were separated by SDS-PAGE and C3b degradation products were then analyzed by Western blotting. CFH bound to serum-resistant *B. burgdorferi* LW2 retained its cofactor activity as shown by the appearance of the characteristic cleavage pattern of C3b (68 and 43 kDa α ´-chain). In contrast, C3b remained intact when *B. garinii* G1 and *B. valaisiana* isolates Bv9, VS116 as well as ZWU3 Ny3 were investigated, in the presence or absence of CFH as evidenced by the presence of the 110 kDa α ´chain and the 75 kDa β-chain (Fig. 5B). Thus, none of the *B. valaisiana* isolates analyzed exhibit an intrinsic proteolytic activity to allow inactivation of the complement component C3b. No complement C3 cleavage products could be detected after incubation with the respective borrelial strains, except for *B. burgdorferi* LW2, showing a fragment that correspond to the 41 kDa α ´chain (Figure S1).

**Figure 6:**
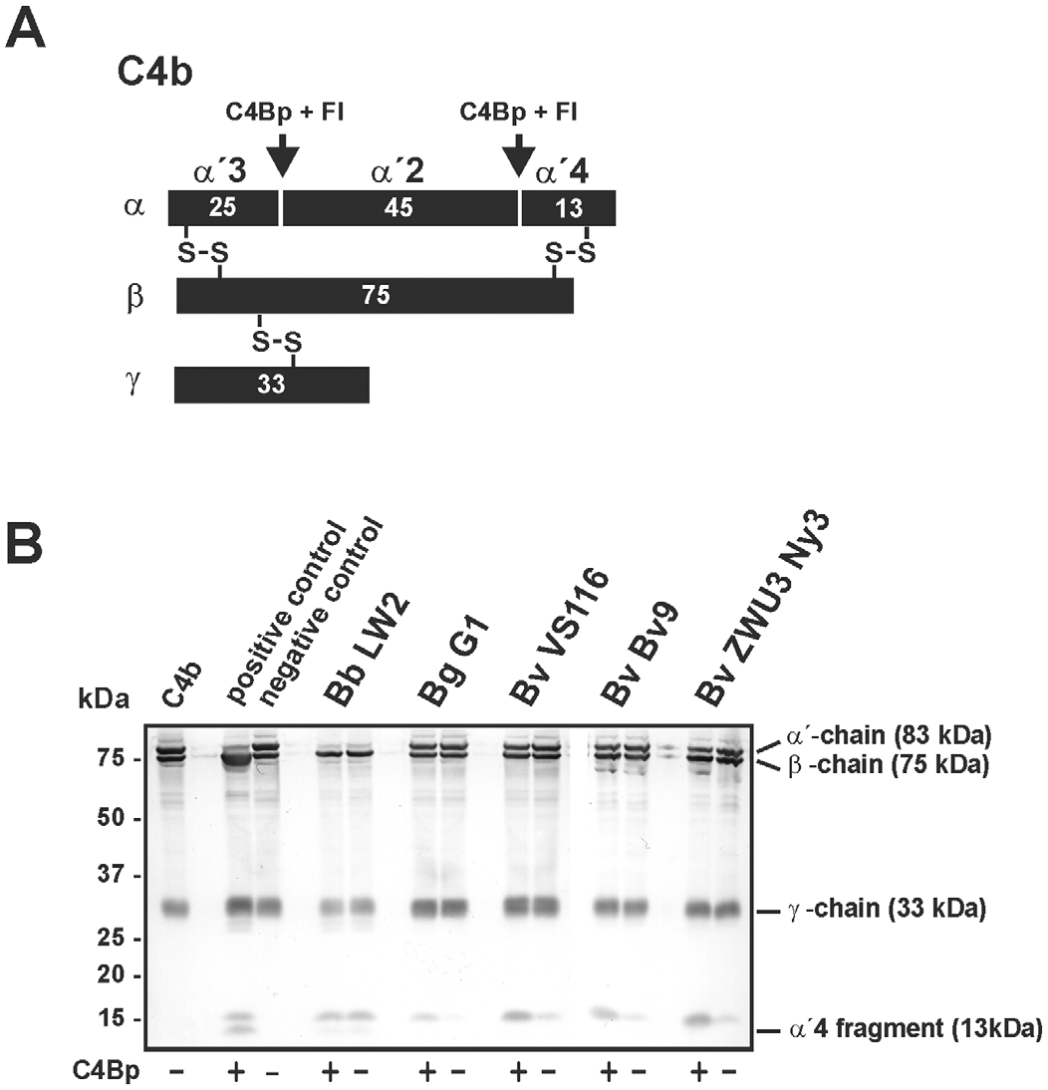
Determination of the C4b proteolytic activity of *B. valaisiana*. (A) Schematic representation of the α-, β-, and the γ-chain of C4b and the cleavage fragments of the α-chain generated by C4Bp and Factor I. (B) Degradation of C4b by an intrinsic proteolytic activity of borrelial cells (5×10^8^) was analyzed by detection of characteristic cleavage fragments after incubation of spirochetes with (+) or without (−) purified C4Bp. *B. burgdorferi* LW2, *B. garinii* G1, *B. valaisiana* isolates Bv9, VS116, and ZWU3 Ny3 were incubated with C4Bp for 60 min at room temperature. After extensive washing with GVB^++^, C4b (1 µg/ml) and Factor I (1 µg/ml) were added and the mixture was incubated for 120 min at 37°C. Subsequently, the samples were heated to 95°C for 5 min, subjected to 10% tris/tricine SDS-PAGE and transferred onto a nitrocellulose membrane. The C4b cleavage products were visualized by Western blotting using a polyclonal goat anti-human C4 antiserum. As a positive control, purified C4b (1 µg) was incubated with C4Bp and Factor I, and as a negative control complement proteins were incubated in the absence of complement regulator C4Bp. The mobility of the α’-, β’-, and γ’-chain and the α’4 fragment is indicated. (+) incubation with all complement proteins; (−) incubation without C4Bp.

Although all borrelial isolates examined were unable to bind complement regulator C4Bp, we sought to determine whether spirochetes, in particular *B. valaisiana* ZWU3 Ny3, are able to cleave C4b in the absence of C4Bp. Thus, cells were incubated with purified C4Bp from human serum, with NHS as a natural source of C4Bp or in GVB^++^ buffer for 60 min before being extensively washed and subsequently purified C4b and purified Factor I were added. To test for C4b degradation, a polyclonal anti-C4 antibody was used that recognizes the 13 kDa α ´4 fragment, the 75 kDa β-chain as well as the 33 kDa γ-chain (Fig 6A). Cleavage of C4b, as detected by visualization of the characteristic α ´4 fragment, was demonstrated only for the positive control but was absent for the borrelial strains analyzed (Fig. 6B). When incubated with C4b purified from human serum alone, or in the absence of C4Bp, the 83 kDa α ´chain remained intact, using *B. garinii* G1, *B. valaisiana* Bv9, VS116, and ZWU3 Ny3 cells (Fig. 6B). In addition, no degradation was found after incubation of these strains with NHS or GVB^++^ buffer (data not shown). Unexpectedly, a weak signal of the C4b cleavage product corresponding to the α ´4 fragment was detectable upon incubation of serum-resistant *B. burgdorferi* LW2 with C4b and Factor I.

Investigating degradation of complement component C4, we found that neither of the Lyme disease spirochetes tested, nor the relapsing fever *B. duttonii* strain LA1 displayed any proteolytic capacity to cleave C4 in either the presence or absence of Factor I (Figure S1).

**Figure 7:**
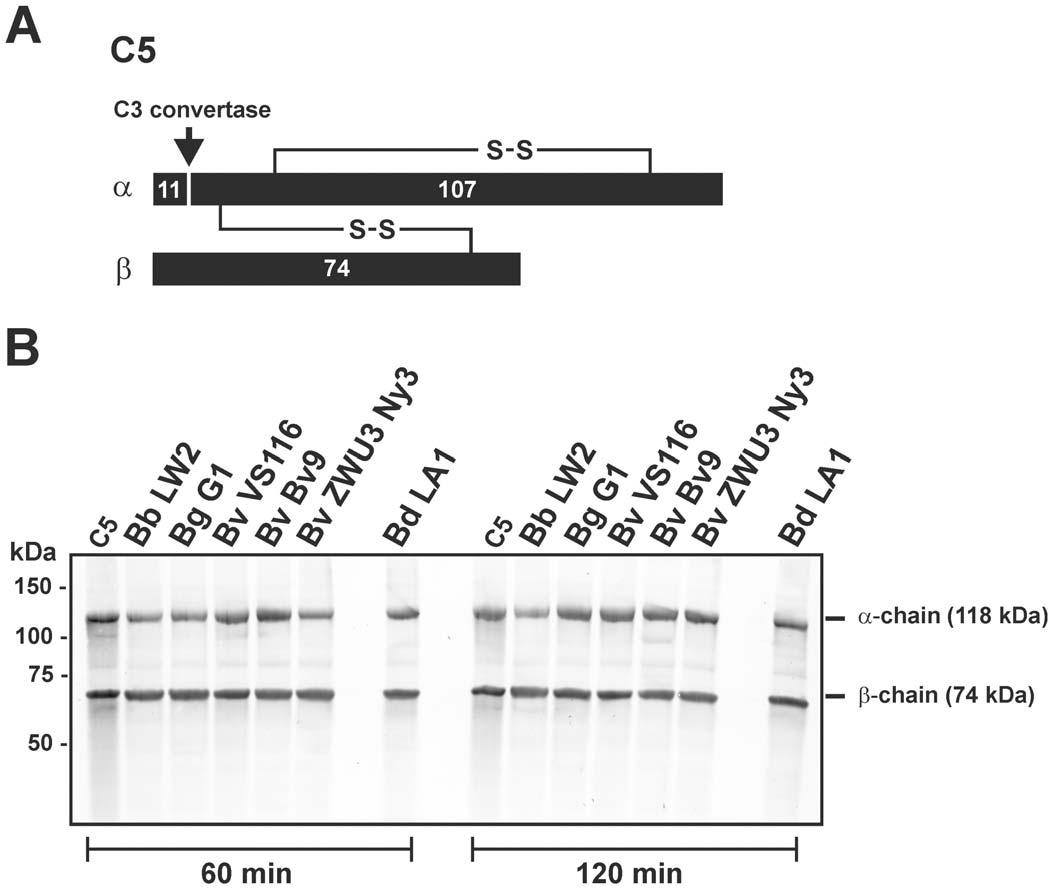
Determination of the C5 proteolytic activity of *B. valaisiana*. (A) Schematic representation of the α-, and β-chain of C5. (B) Degradation of C5 by an intrinsic proteolytic activity of spirochetes (5×10^8^) was analyzed by detection of potential cleavage products after incubation of cells with purified C5. *B. burgdorferi* LW2, *B. garinii* G1, *B. valaisiana* isolates Bv9, VS116, ZWU3 Ny3, and *B. duttonii* LA1 were incubated with 1 µg C5 for 60 min and 120 min at 37°C. After centrifugation, supernatants were subjected to 10% tris/tricine SDS-PAGE and transferred onto a nitrocellulose membrane. The C5 fragments were visualized by Western blotting using a polyclonal goat anti-human C5 antiserum. As a negative control, purified C5 (1 µg) was incubated under the same conditions. The mobility of the 118 kDa α-chain and the 74 kDa β-chain is indicated.

In order to test the inactivation capacity on complement component C5, spirochetes were incubated with purified C5 for 60 and 120 min. After centrifugation, supernatants were subjected to SDS-PAGE and C5 cleavage products were analyzed by Western blotting using a goat anti-C5 antibody. No degradation fragments of the 118-kDa α-chain or of the 74-kDa β-chain were detected indicating that spirochetes lacked proteolytic activity for the cleavage of C5 (Fig. 7).

Next, to exclude proteolytic degradation of complement components C3, C3b, C4, C4b, and C5 by secreted proteases we also analyzed supernatants collected from late logarithmic borrelial cultures. Spirochete-free supernatants were incubated with 2 µg of purified complement components for 180 min at 37°C. After termination of the reaction, a portion of the reaction mixtures were applied to SDS-PAGE and cleavage products were detected by Western blotting. As shown in Figure S2, no degradation fragments could be visualized, suggesting that spirochetes did not secrete proteases into the culture medium.

Taken together, these findings strongly suggest that serum-resistant *B. valaisiana* ZWU3 Ny3 displayed no intrinsic proteolytic activity that would have resulted in inhibition of complement by cleavage of C3, C3b, C4, C4b, and C5.

### Determination of the C3a and C4a Generation by *B. valaisiana*

**Figure 8:**
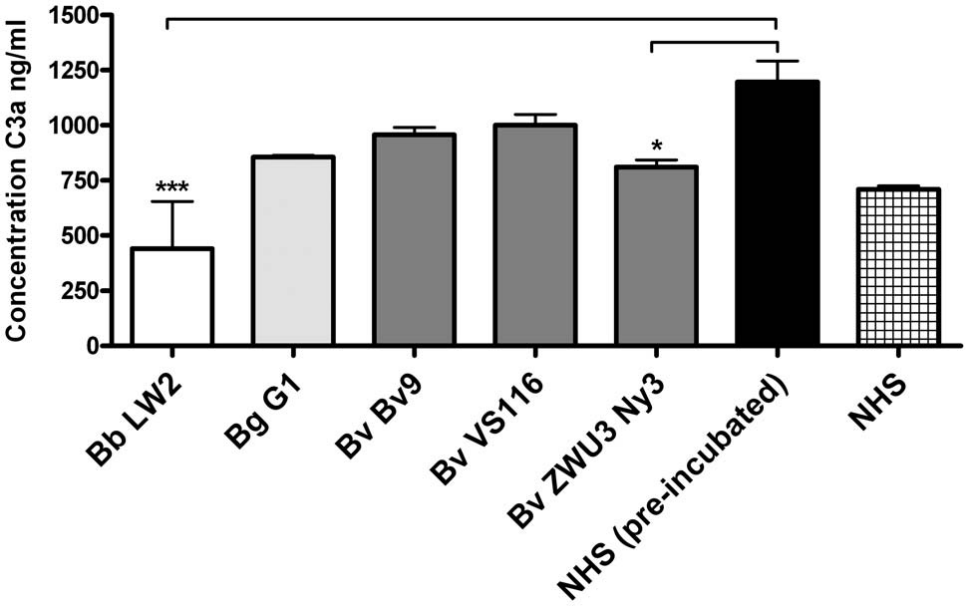
Determination of the C3a generation by *B. valaisiana*. Spirochetes (6×10^6^) were incubated with 25% of NHS for 5 min at 21°C and activation of C3 was then analyzed by the MicroVue C3a Plus ELISA. Generated C3a was detected using a monoclonal anti-C3a capture antibody and a HRP-conjugated polyclonal anti-C3 antiserum. All experiments were performed three times with at least three replicates, obtaining very similar results. For clarity, data of a representative experiment are shown. Error bars represent ± SD. Raw data were analyzed by one-way ANOVA with post hoc Bonferroni correction. ***p<0.001; *p<0.05.

To further evaluate the ability of serum-resistant *B. burgdorferi* LW2 and *B. valaisiana* ZWU3 Ny3 to inhibit complement activation at the level of C3 and C4 we sought to quantify the generation of C3a and C4a after incubation of spirochetes with human serum by ELISA. As demonstrated in figure 8, C3 activation was significantly reduced for *B. burgdorferi* LW2 (p-value <0.001) as well as for *B. valaisiana* ZWU3 Ny3 (p-value 0.05) while, in contrast, a stronger C3a generation could be detected for serum-sensitive *B. garinii* G1 as well as *B. valaisiana* Bv9 and VS116. Of note, pre-incubation of NHS for 5 min at 21°C resulted in a somewhat higher C3a generation compared to untreated NHS samples. Nevertheless, the data collected support the notion that ZWU3 Ny3 inhibits C3 activation to some extent.

To investigate C4a generation, spirochetes were incubated with 2% or 25% human serum for 5 min at 37°C and C4a was measured using ELISA. However, no significant differences could be observed between the serum-resistant and serum-sensitive strains analyzed (data not shown).

## Discussion

Recruitment of complement regulators of the alternative pathway, CFH and FHL1 by serum-resistant *B. burgdorferi*, *B. afzelii*, and *B. spielmanii* enable these spirochetes to inhibit complement activation at the central step of the cascade (C3 level), and thereby overcome complement-mediated killing [24], [25], [29], [31], [36]. Here, we demonstrate that *B. valaisiana* isolates differ in their susceptibility to human serum. Similar to the serum-sensitive *B. garinii* control strain G1, *B. valaisiana* isolate Bv9 and *B. valaisiana* type strain VS116 strongly activate complement and large amounts of complement components were deposited on their surface, whereas isolate ZWU3 Ny3 resists complement-mediated killing and showed limited or no deposition of destructive complement components. Irrespective of whether they displayed a serum-sensitive or resistant phenotype, all three *B. valaisiana* isolates acquired CFHR-1 and strain Bv9, in addition, also bound CFHR2 from human serum. Despite binding of these complement regulators, Bv9 and VS116 failed to regulate complement activation, thus permitting bacteriolysis as previously shown for *B. lusitaniae* [49]. More interestingly, isolate ZWU3 Ny3 bound neither complement regulators of the alternative pathway CFH and FHL1, nor of the classical and lectin pathways, C4Bp and C1-Inh, respectively. We also showed that none of the *B. valaisiana* isolates displayed an intrinsic proteolytic capacity to inactivate C3, C3b, C4, C4b, and C5. These findings strongly suggest that *B. valaisiana* ZWU3 Ny3 resist complement-mediated killing by a novel mechanism of immune evasion.

It has recently been shown that spirochetes of the *B. burgdorferi* sensu lato-complex exhibit a high diversity in their susceptibility pattern to human complement [23], [29], [46], [47], [49], [53]–[56]. Concerning serum susceptibility of *B. valaisiana*, the isolates investigated so far showed a serum-sensitive phenotype [53], [56], [57]. Due to the fragmentary information available and the limited number of isolates (n = 5) tested so far, the question of whether this particular genospecies, in general, exhibits a serum-sensitive phenotype, as observed for *B. garinii* and *B. lusitaniae* [46], [47], [49], [53], [56], has not been adequately addressed. Here, we show for the first time that a particular isolate of *B. valaisiana* ZWU3 Ny3 may resist complement-mediated killing even though the majority of isolates are susceptible to human serum. Hence, it is tempting to speculate that complement-resistant cells are protected from innate immune defenses and *qua argumentum e contrario* can more efficiently establish an infection and survive in the human host. Although *B. valaisiana* has not been successfully isolated from patients yet, there is some evidence that this particular genospecies is an, albeit infrequent, cause of Lyme disease [3], [4], [6]. However, to which extent serum-resistant *B. valaisiana* are responsible for establishing human infections remains unclear and necessitates cultivation of this particular genospecies from Lyme disease patients.

To evade complement-mediated killing, serum-resistant spirochetes acquire complement regulators CFH and FHL1 which in turn leads to an efficient inactivation of deposited C3b [24]–[26], [29], [53], [55]. In addition, no or inadequate binding of CFH and/or FHL1 is correlated with an excess of complement activation and deposition of harmful complement components on the borrelial surface as demonstrated for *B. garinii* and *B. lusitaniae* [24], [46], [47], [49], [53], [56]. To our knowledge, binding of human complement regulators to viable *B. valaisiana* cells has not been reported so far, although a proposed CFH-binding protein has recently been identified using cell lysates of isolate VS116 [58]. Here, we show for the first time that a member of the *B. valaisiana* genospecies did not bind the key complement regulators of the alternative, classical, and lectin pathways (CFH, FHL1, C4Bp as well as C1-Inh) (Figure 4). Interestingly, all *B. valaisiana* isolates were able to bind CFHR1 and isolate Bv9 also bound CFHR2, irrespective of their serum susceptibility. More recently, we showed that surface-bound CFHR1 and CFHR2 were insufficient to completely prevent complement activation, and thus failed to protect spirochetes from complement-mediated killing [44], [49], [50]. In line with this assumption, large amounts of C3, C4, C6 and MAC were detectable on the spirochetal surface following incubation of serum-sensitive *B. valaisiana* VS116 and Bv9 with active human serum (Figure 2). In contrast, the majority of cells of *B. valaisiana* ZWU3 Ny3 did not show strong deposition of lethal complement components akin to the serum-resistant *B. burgdorferi* control strain LW2, indicating that this particular isolate uses other mechanisms to combat complement activation.

By investigating the borreliacidal effect of complement on *B. valaisiana,* we observed a complete loss of cell mobility after serum incubation and excessive bleb formation for isolates VS116 and Bv9, (Figure 1 and 2) which is in complete agreement with previous findings showing that *B. valaisiana* is highly sensitive to human complement [53], [56]. In contrast, the serum-resistant isolate *B. valaisiana* ZWU3 Ny3 and the control strain *B. burgdorferi* LW2 showed continuous growth in human serum and limited complement deposition on their surface. Our observation of the borreliacidal effect of human complement on spirochetes is in accordance with previous findings that serum-sensitive *B. valaisiana*, *B. garinii,* and *B. lusitaniae* were strongly affected by complement [47], [49], [56].

Serum resistance of spirochetes is primarily mediated by the interaction of CFH and FHL1 with two distinct borrelial outer surface proteins, namely CspA and CspZ [26]–[30], [35], [36]. Additionally, spirochetes that lack the CspA or CspZ encoding genes through loss of the respective plasmids or by inactivation of the *cspA* gene become serum-sensitive [41]–[43]. Binding of CFHR1 and CFHR2 also necessitates interacting ligands, which have previously been identified to be ErpP (CRASP-3), ErpC (CRASP-4) and ErpA (CRASP-5), all of which belong to the polymorphic OspE protein family [28], [30], [44], [50], [59]. Due to the considerable degree of identity between the C-terminal domains of CFHR1 (100 and 97%) and CFHR2 (89 and 61%) to the C-terminal SCRs 19 and 20 of CFH [16], ErpP (CRASP-3), ErpC (CRASP-4), ErpA (CRASP-5) as well as OspE orthologs of *B. burgdorferi* also bind complement regulator CFH [28]. Further analyses revealed that whereas recombinant CRASP-3, CRASP-4, and CRASP-5 readily bind CFH, the native proteins predominately interacted with CFHR1 and CFHR2 and failed to bind CFH [44], [50]. Employing ligand affinity blotting, we identified potential CFH/CFHR1-binding proteins of *B. valaisiana* isolates Bv9 and VS116 with an estimated molecular weight of ∼15 kDa (Figure 3) which in regard to their CFH/CFHR1-binding capabilities strongly resemble the OspE orthologs of *B. lusitaniae* and *B. spielmanii,* as well as a ∼17 kDa protein recently described for *B. valaisiana* VS116 [49], [58], [60]. While the denaturated ∼17 kDa protein of *B. valaisiana* VS116 described by Bhide et al. [58] potentially bind CFH in a ligand affinity blot, the authors did not analyzed binding of CFH to viable spirochetes, strongly suggesting that the native protein displays a different binding capability to this complement regulator. In conclusion, it is apparent, that the findings from our binding analysis with viable cells better serve to explain the serum susceptible phenotype of a given borrelial isolate than do conclusions based on ligand affinity blots.

Pathogenic microorganisms develop different strategies to counteract complement [61]–[64]. Apart from the acquisition of CFH and FHL1, Lyme disease spirochetes appear to bind the negative regulator of the classical pathway, C4Bp [65]. Employing different methodologies and conditions, we were unable to detect binding of this regulator to *B. valaisiana*, *B. burgdorferi*, and *B. garinii* (Figure 4). Under identical experimental conditions, binding of C4Bp could be demonstrated for the relapsing fever *B. duttonii* strain LA1 which is in line with previously published data [52], [66]. However, binding of C4Bp failed to protect serum-sensitive *B. garinii* from the destructive attack of complement, thus the biological impact of the interaction of this regulator with Lyme disease spirochetes for complement evasion is elusive.

Serum-resistant *B. valaisiana* ZWU3 Ny3 deposited limited amounts of C3 and C4 on the surface (Figure 2) suggesting that complement activation was influenced at very early steps of the cascade. Complement inhibition should be accompanied by a reduced release of potent bioactive mediators C3a and C4a upon activation. The activation assays investigated (Figure 8) showed a statistically significant reduction of C3 activation upon pre-incubation of NHS with serum-resistant *B. valaisiana* ZWU3-Ny3 (p-value<0.05) and serum-resistant *B. burgdorferi* LW2 (p-value <0.001) while no reduction could be observed concerning the generation of C4a. The high amount of C4a generated by pre-incubation of NHS might account for the similar C4a values calculated for all borrelial strains and, seemingly, did not mirror the finding obtained by immunofluorescence microscopy. Thus, in light of these experiments it is not entirely clear how this particular *B. valaisiana* strain negatively influences complement activation at the level of C4.

Excluding CFH, FHL1, and C4Bp as potential interacting molecules responsible for mediating serum resistance of ZWU3 Ny3, we also investigated binding of C1-Inh as an additional promising candidate. By analyzing binding of serum-derived regulators, we detected binding of C1-Inh exclusively to the tick-borne relapsing fever *B. duttonii* strain LA1, but not to Lyme disease spirochetes, including isolate ZWU3 Ny3 (Figure 4), indicating that this specific isolate exerts different means to resist complement-mediated killing.

Concerning degradation of key complement components, diverse microorganisms secrete to inactivate complement [67]–[71]. Investigating degradation of C3, C3b, C4, C4b as well as C5, none of the key components of the complement cascade were cleaved by serum-resistant *B. valaisiana* ZWU3 Ny3 or could be detected in the supernatant of this strain (Figures 5, 6, and 7, and Figures S1 and S2), suggesting that this isolate lacks potent proteolytic enzymes to inactivate complement.

In conclusion, we demonstrated that *B. valaisiana* isolates differ in their susceptibility to human serum, thus providing some evidence that in particular serum-resistant isolates might cause Lyme disease. Contrary to our expectations, certain *B. valaisiana* isolates appear to possess different molecular mechanism(s) to inhibit complement activation, independently of the recruitment of complement regulators or by inactivation of central complement components. Even though that we are currently unable to decipher the precise molecular mechanism, it is tempting to speculate that *B. valaisiana* ZWU3 Ny3 expresses an outer surface protein that directly interacts with components of the complement system to inhibit complement activation. Further investigation is required to identify potential complement inhibitory protein(s) of this particular borrelial strain.

## Supporting Information

Figure S1 **Determination of the C3 and C4 proteolytic activity of** ***B. valaisiana.*** Degradation of C3 (A) and C4 (B) by an intrinsic proteolytic activity of borrelial cells (5×10^8^) was analyzed by detection of characteristic cleavage fragments after incubation of spirochetes. *B. burgdorferi* LW2, *B. garinii* G1, *B. valaisiana* isolates Bv9, VS116, and ZWU3 Ny3 were incubated with C3 or C4 for 120 min at 37°C. Subsequently, the samples were heated to 95°C for 5 min, subjected to 10% tris/tricine SDS-PAGE under reducing conditions. The C3 and C4 cleavage products were visualized by Western blotting using a polyclonal goat anti-human C3 and a polyclonal goat anti-human C4 antiserum, respectively. As a control, purified C3 or C4 (1 µg each) were incubated under identical conditions. The mobility of the C3 and C4 cleavage fragments are indicated and the mobility of the protein standard is indicated on the left. The mobility of the marker proteins is indicated on the left.
(TIF)

Figure S2 **Determination of complement degradation by external proteases.** Supernatants obtained from late logarithmic cultures (10 µl each), as well as BSK medium (negative control) were incubated with 2 µg of purified complement components for 180 min at 37 °C. As a further control, purified C3, C3b, C4, C4b, and C5 were incubated under identical conditions.The reaction mixtures were terminated by adding SDS sample buffer and one fourth of the reactions were subjected to 10% tris/tricin SDS-PAGE under reducing conditions. After transfer to nitrocellulose membranes, complement components were detected by using the appropriate polyclonal antisera as described in the Material & Method section. The mobility of the marker proteins is indicated on the left. For detection of complement components in culture medium, same amounts of BSK were subjected to 10% tris/tricine SDS-PAGE and proteins were transferred to nitrocellulose membranes. Complement components were then detected using appropriate polyclonal antibodies. The mobility of marker proteins is indicated on the left.
(TIF)
