# Supplementary figures and images for: Borrelia valaisiana Resist Complement-Mediated Killing Independently of the Recruitment of Immune Regulators and Inactivation of Complement Components
Source: PLoS One. 2013 Jan 8;8(1):e53659. doi: 10.1371/journal.pone.0053659 (PMC3539980; doi:10.1371/journal.pone.0053659)

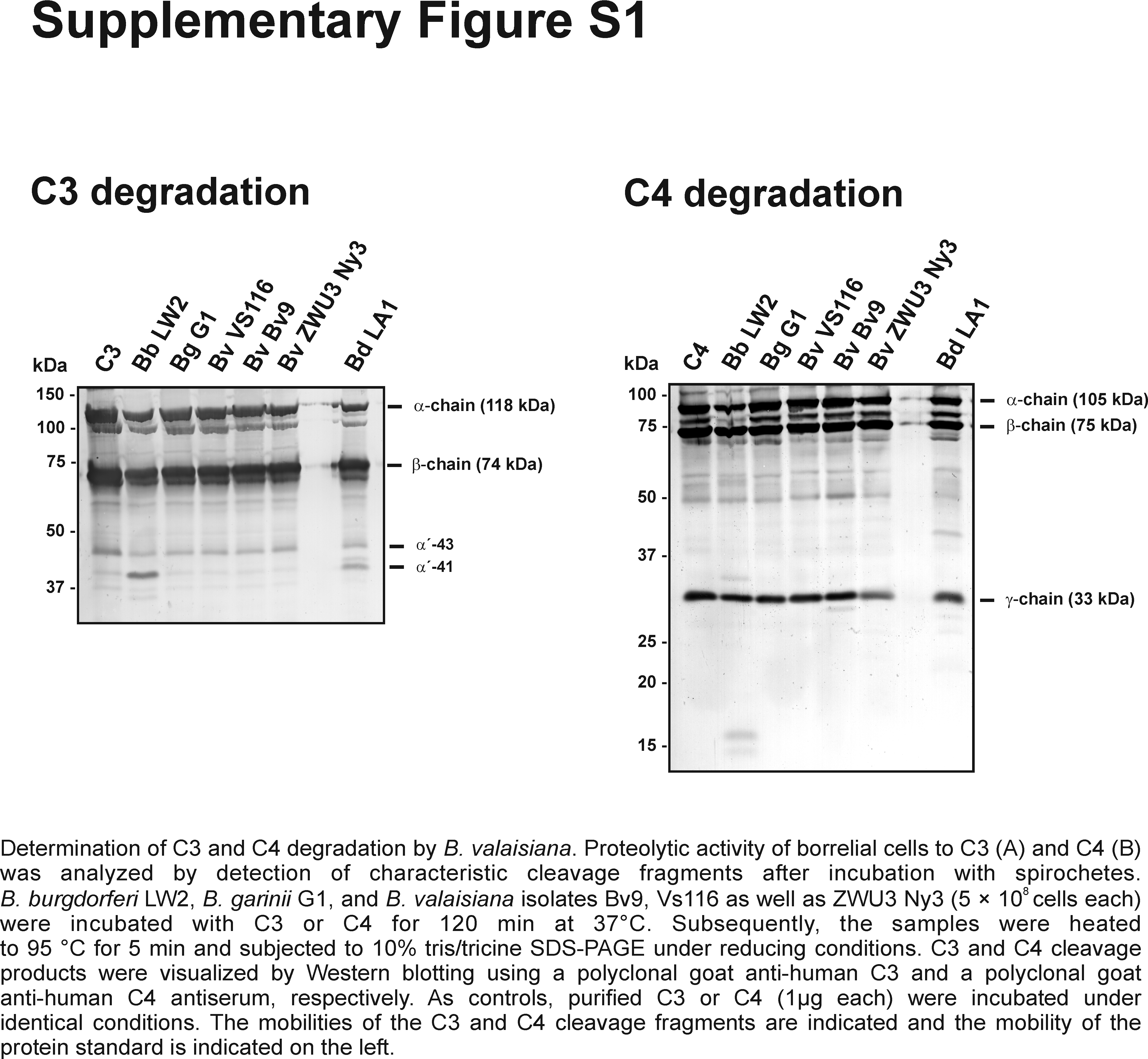

Supplement: Figure S1 — Determination of the C3 and C4 proteolytic activity of B. valaisiana. Degradation of C3 (A) and C4 (B) by an intrinsic proteolytic activity of borrelial cells (5×10^8^) was analyzed by detection of characteristic cleavage fragments after incubation of spirochetes. B. burgdorferi LW2, B. garinii G1, B. valaisiana isolates Bv9, VS116, and ZWU3 Ny3 were incubated with C3 or C4 for 120 min at 37°C. Subsequently, the samples were heated to 95°C for 5 min, subjected to 10% tris/tricine SDS-PAGE under reducing conditions. The C3 and C4 cleavage products were visualized by Western blotting using a polyclonal goat anti-human C3 and a polyclonal goat anti-human C4 antiserum, respectively. As a control, purified C3 or C4 (1 µg each) were incubated under identical conditions. The mobility of the C3 and C4 cleavage fragments are indicated and the mobility of the protein standard is indicated on the left. The mobility of the marker proteins is indicated on the left. (TIF) [file pone.0053659.s001.tif]

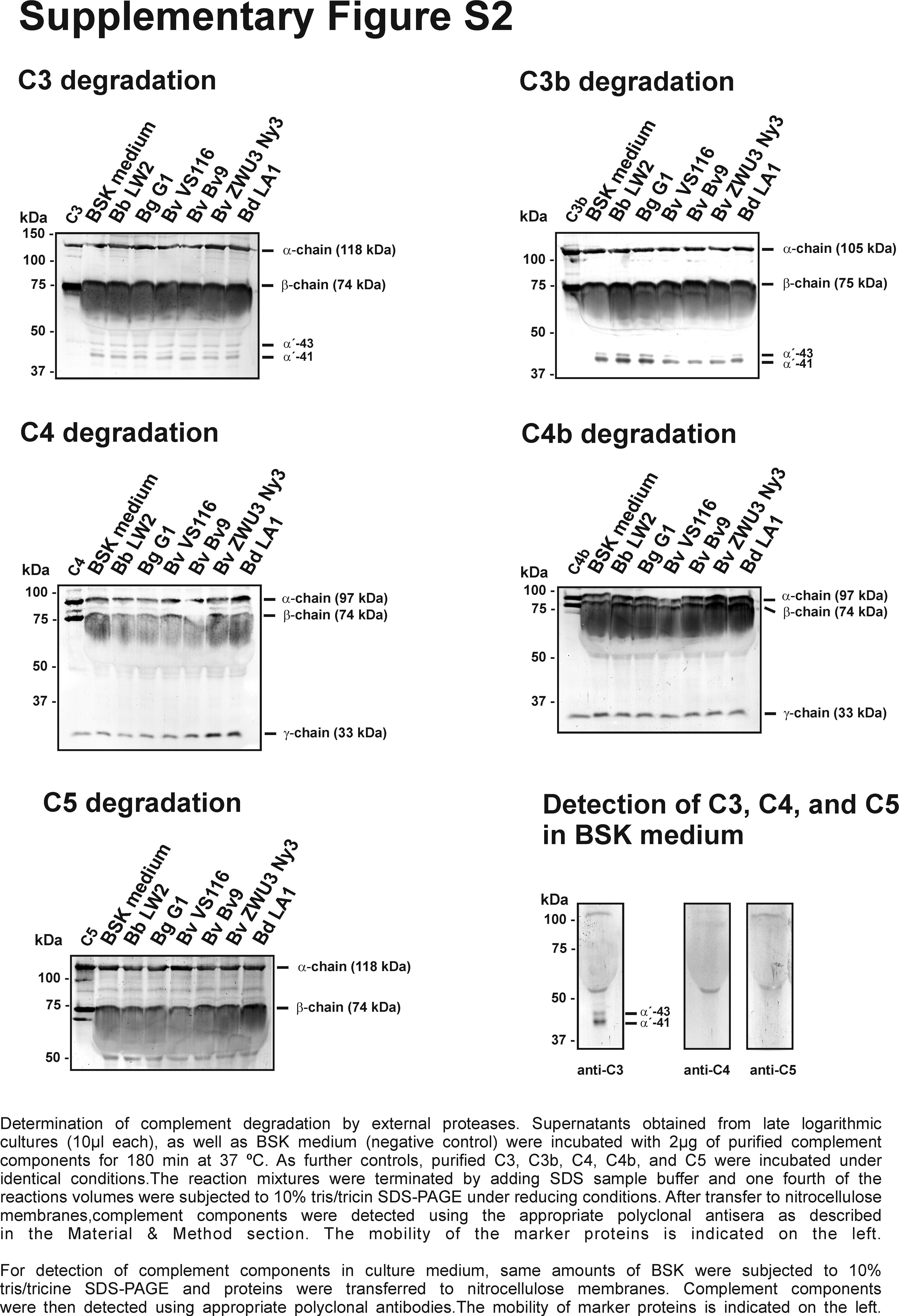

Supplement: Figure S2 — Determination of complement degradation by external proteases. Supernatants obtained from late logarithmic cultures (10 µl each), as well as BSK medium (negative control) were incubated with 2 µg of purified complement components for 180 min at 37 °C. As a further control, purified C3, C3b, C4, C4b, and C5 were incubated under identical conditions.The reaction mixtures were terminated by adding SDS sample buffer and one fourth of the reactions were subjected to 10% tris/tricin SDS-PAGE under reducing conditions. After transfer to nitrocellulose membranes, complement components were detected by using the appropriate polyclonal antisera as described in the Material & Method section. The mobility of the marker proteins is indicated on the left. For detection of complement components in culture medium, same amounts of BSK were subjected to 10% tris/tricine SDS-PAGE and proteins were transferred to nitrocellulose membranes. Complement components were then detected using appropriate polyclonal antibodies. The mobility of marker proteins is indicated on the left. (TIF) [file pone.0053659.s002.tif]
